# Incidence of Multidrug-resistant Tuberculosis in Sindh, Pakistan

**DOI:** 10.7759/cureus.4571

**Published:** 2019-04-30

**Authors:** Rashid A Khan, Anwar A Shaikh, Ghulam Q Bulaadi

**Affiliations:** 1 Pulmonology, Civil Hospital, Jamshoro, PAK; 2 Radiology, Civil Hospital, Jamshoro, PAK

**Keywords:** tuberculosis, resistance, pakistan, incidence, multidrug-resistant tb

## Abstract

Introduction

Pakistan carries a high burden of pulmonary tuberculosis. Resistance to anti-tuberculosis drugs is increasing rapidly in this region. The purpose of this study is to determine the incidence of multidrug-resistant tuberculosis (MDR-TB) in Sindh, Pakistan.

Methods

We conducted a prospective, observational study in the department of pulmonology at Civil Hospital, Jamshoro. A total of 169 patients with smear-positive sputum after two months of compliant therapy with first line anti-tuberculosis therapy (ATT) were included in the study. Drug susceptibility testing was performed; this involved the preparation and testing of a concentration series of drugs against the Mycobacterium (M.) tuberculosis complex. Data were analyzed using Statistical Package for the Social Sciences (SPSS) software Version 18.0 (SPSS Inc., Chicago, US).

Results

Overall, the frequency of MDR-TB patients being treated with first-line ATT was 40.2% (68/169). Among these cases of MDR-TB, there were 36 (53%) men and 32 (47%) women. The most common second-line drug resistance was to ofloxacin (43.2%; n=73).

Conclusions

The growing drug resistance of M. tuberculosis is a problem in Pakistan, and measures should be taken to reduce the incidence of resistance, including increasing patient compliance to therapy.

## Introduction

Tuberculosis (TB) has been a global health concern for decades. Although a large amount of work has been done to eradicate TB, in 2014, more than nine million new cases of TB surfaced - almost one and a half million of these proved fatal [1]. Presently, there are 22 high-TB-burden countries that account for almost 80% of the world’s TB cases. Most of the documented data from these high burden countries do not reflect the actual figures due to the improper implementation of data collection processes, inefficient surveillance systems, and incomplete coverage [2].

Multidrug-resistant tuberculosis (MDR-TB), resistant to both isoniazid and rifampicin, is a major health concern, as it poses tremendous challenges for not only the public health sector but also the pulmonologists, as well as the general physicians of Pakistan, who are managing TB [3]. According to the World Health Organization (WHO) statistics from 2012, the incidence rate of TB in Pakistan is 231/100,000 people and the prevalence rate is 376/100,000 people. Furthermore, of all the pulmonary TB cases identified in 2012, 3,700 were MDR-TB. Additionally, 55/1,602 were laboratory-proven MDR retreatment cases [4].

Various studies have been conducted to assess the risk factors behind the increasing ratio of MDR-TB. Previous TB history, a history of multiple antituberculosis therapy (ATT) courses, prescriptions from multiple clinicians, unsupervised general practitioner management, age <45 years, male gender, immigration from another area with TB endemic/resistant strains, unemployment, and below satisfactory living conditions were associated with an increased risk of developing MDR-TB. Patients with evidence of the failure of the first-line ATT, patients who defaulted appointments, and patients who relapsed during the treatment are also at a high risk of developing MDR-TB [5-6].

Akhtar et al. reported MDR-TB was more prevalent at the pulmonary site (68%), in the reproductive age group (57%), and in the urban areas (97%) [7] of Punjab, Pakistan. In a meta-analysis in India, 46.9% of the patients had TB resistance to isoniazid, 27.6% to rifampicin, 33.7% to ethambutol, and 34.7% to streptomycin in retreatment cases [8]. Although data are available for other provinces of Pakistan, there is still not enough data available about MDR-TB in Sindh. The purpose of this study is to determine the incidence of MDR-TB in Sindh, Pakistan.

## Materials and methods

A prospective observational study was conducted from January 1, 2017, to December 31, 2017, in the Civil Hospital Department of Pulmonology, in Jamshoro, Pakistan.

Patients aged 18 years and older registered for TB management in the outpatient department were recruited. Patients who had been taking a first-line anti-TB regimen for a minimum of two months and were still positive for Mycobacterium tuberculosis (MTB), either on sputum smears or culture, were included. Informed consent was provided by all patients. The study was approved by the institutional review board.

Exclusion criteria included patients who had become negative for MTB on sputum smear or culture within two months of first-line ATT, sputum-positive patients who were non-adherent to therapy, and patients who refused to participate. Drug adherence was confirmed from the patient’s TB drug and dosage card.

Culture and sensitivity were assessed using a sputum sample or, in cases of an absent sputum sample, from bronchoalveolar lavage. Isolation of Mycobacterium from the specimens was achieved by Lowenstein-Jensen medium and Mycobacterium Growth Indicator Tube medium (Becton Dickinson, Franklin Lakes, NJ, USA). After that, MTB was isolated using the BACTEC NAP test (Becton Dickinson,). The agar proportion method on enriched Middle brook 7H10 medium (BBL Microbiology Systems, Cockeysville, MD, USA) was utilized for testing drug susceptibility. The drug concentrations utilized were as follows: isoniazid 0.2μg/ml, rifampicin 1μg/ml, ethambutol 5μg/ml, and streptomycin 2μg/ml and 10μg/ml. For pyrazinamide sensitivity, BACTEC 7H12 medium was used at pH 6.0 at 100μg/ml (BACTEC PZA test medium, Becton Dickinson).

MDR was labeled for MTB strains that were resistant to both isoniazid and rifampin. Only then were MDR strains tested for sensitivity to second-line line ATT at the following concentrations: capreomycin 10μg/ml, ofloxacin 2μg/ml, ethionamide 5μg/ml, and kanamycin 6μg/ml.

The patients completed a brief questionnaire requesting patient demographics such as age, gender, and history of TB contact. Data were analyzed using the Statistical Package for the Social Sciences (SPSS) software Version 18.0 (Chicago, SPSS Inc.).

## Results

A total of 169 participants were enrolled in this study. The mean ± standard deviation (SD) age of participants was 26.23 ± 12.45 years. There were 93 women (55%) and 76 men (45%) in the study. There were 117 (69.2%) patients who had a positive history of household TB contact.

Sensitivity and resistance to first-line ATT were analyzed. Most of the TB cases (n=113; 66.9%) were resistant to isoniazid but were sensitive to ethambutol (n=139; 82.8%). There were 40.2% (n=68) cases of MDR-TB. All these patients were being treated with first-line ATT. Among the cases of MDR-TB, there were 36 (53%) men and 32 (47%) women. The sensitivity and resistance patterns for first-line ATT is shown in Table 1.

**Table 1:** Resistance to first-line antituberculosis therapy (n=169) Abbreviations: MDR, multidrug resistance

Drug	Sensitivity n (%)	Resistance n (%)
Streptomycin	98 (57.9%)	71 (42.1%)
Isoniazid	56 (33.1%)	113 (66.9%)
Rifampicin	91 (53.85%)	78 (46.15%)
Isoniazid + rifampicin (MDR)	46 (27.2%)	68 (40.2%)
Pyrazinamide	93 (55%)	76 (45%)
Ethambutol	139 (82.8%)	30 (17.8%)

Regarding the second-line therapies, many TB cases (n=73; 43.2%) were resistant to ofloxacin while most were sensitive to kanamycin (n=165; 97.68%). The sensitivity and resistance patterns for second-line ATT are shown in Table 2.

**Table 2:** Resistance to second-line antituberculosis therapy (n=169)

Drug	Sensitivity n (%)	Resistance n (%)
Kanamycin	165 (97.6%)	4 (2.4%)
Capreomycin	166 (98.2%)	3 (1.8%)
Ethionamide	155 (91.7%)	14 (8.3%)
Ofloxacin	96 (56.8%)	73 (43.2%)

As one of the more common factors leading to resistance, patient compliance with therapy was recorded. One hundred thirty-nine patients (82.2%) were taking their medications regularly, 28 patients (16.6%) were taking their medications irregularly, and two patients (1.2%) were not taking their medications at all.

## Discussion

Ineffective measures to detect, diagnose, treat, and eradicate TB facilitates an increase in the emergence of MDR-TB [9]. Countries such as Pakistan that are already facing emergencies such as humanitarian crises and conflicts pay little attention to this rising health concern [10]. There is broad data available from Pakistan that report a rising incidence of not only MDR-TB but also extensively drug-resistant TB strains [11]. Our study reported an MDR-TB incidence of 40.2%; 53% of these were men and 47% were women.

Hasan et al. reported that in the last 17 years, the incidence of MDR-TB has increased from 0% to 34.3% in women and from 5.5% to 32% in men [11]. Akhtar et al. reported that 69% of the cases were identified as MDR-TB and significantly associated with re-treatment. In addition, 41% of the cases showed resistance to all first-line ATT and 28% had resistance to oral first-line ATT. Furthermore, 42% of the cases were resistant to fluoroquinolones and 5% to ethionamide [7]. Our results are similar, with 40% MDR-TB, 42% resistant to streptomycin, 67% resistant to isoniazid, 46% resistant to rifampicin, 45% to pyrazinamide, and 18% to ethambutol. For the second-line of treatment, ofloxacin resistance was 43%, ethionamide 8%, and kanamycin and capreomycin had the lowest resistance rate with almost 2% each. In an Iranian study, the frequency of MDR-TB was 15% [6]. Another local study, conducted in 2010, reported the MDR prevalence to be 5%, with a significant association with female gender and a history of previous TB treatment [12].

The inappropriate management of drug-resistant TB, such as using poor-quality second-line ATT, relying on a single agent, inefficient drug combinations, and failing to ensure drug adherence, are probable causes of the increase in the incidence of MDR-TB [13]. In our study, 82.2% of the participants were not adherent to their drug regimens.

This study has several limitations. Patients were only tested for strain resistance to four of the six second-line drugs. Additionally, the study size was small and may not accurately reflect the entire population.

The alarming results of this study emphasize the urgent need for conducting population-based nationwide drug resistance surveys. It further emphasizes the immediate need to take actions to make the patients more adherent and compliant with their tuberculosis treatment.

## Conclusions

The incidence of MDR-TB is alarmingly high. The amplification of TB awareness and management programs, along with rigorous infection control measures, are crucial for putting an end to this emerging health concern. MDR-TB detection and awareness programs must be of high quality, and treatments should be based on globally recommended regimens such as WHO guidelines.
